# T-Cell Immunophenotyping Distinguishes Active From Latent Tuberculosis

**DOI:** 10.1093/infdis/jit265

**Published:** 2013-09-15

**Authors:** Katrina M. Pollock, Hilary S. Whitworth, Damien J. Montamat-Sicotte, Lisa Grass, Graham S. Cooke, Moses S. Kapembwa, Onn M. Kon, Robert D. Sampson, Graham P. Taylor, Ajit Lalvani

**Affiliations:** 1 Tuberculosis Research Centre, Department of Respiratory Medicine, National Heart and Lung Institute, Imperial College London, United Kingdom; 2 Section of Infectious Diseases, Department of Medicine, Imperial College London, United Kingdom; 3 Department of GU and HIV Medicine, The North West London Hospitals NHS Trust, Northwick Park Hospital, Harrow, United Kingdom; 4 Tuberculosis Service, St Mary's Hospital, Imperial College Healthcare Trust, London, United Kingdom; 5 Centre for Respiratory Infection, Flow Cytometry Facility, National Heart and Lung Institute, Imperial College London, United Kingdom

**Keywords:** *Mycobacterium tuberculosis*, HIV, latent tuberculosis infection, active tuberculosis, biomarker

## Abstract

***Background.*** Changes in the phenotype and function of *Mycobacterium tuberculosis* (M. tuberculosis)-specific CD4^+^ and CD8^+^ T-cell subsets in response to stage of infection may allow discrimination between active tuberculosis and latent tuberculosis infection.

***Methods.*** A prospective comparison of M. tuberculosis-specific cellular immunity in subjects with active tuberculosis and latent tuberculosis infection, with and without human immunodeficiency virus (HIV) coinfection. Polychromatic flow cytometry was used to measure CD4^+^ and CD8^+^ T-cell subset phenotype and secretion of interferon γ (IFN-γ), interleukin 2 (IL-2), and tumor necrosis factor α (TNF-α).

***Results.*** Frequencies of CD4^+^ and CD8^+^ cells secreting IFN-γ-only, TNF-α-only and dual IFN-γ/TNF-α were greater in active tuberculosis vs latent tuberculosis infection. All M. tuberculosis-specific CD4^+^ subsets, with the exception of IL-2-only cells, switched from central to effector memory phenotype in active tuberculosis vs latent tuberculosis infection, accompanied by a reduction in IL-7 receptor α (CD127) expression. The frequency of PPD-specific CD4^+^ TNF-α-only-secreting T cells with an effector phenotype accurately distinguished active tuberculosis from latent tuberculosis infection with an area under the curve of 0.99, substantially more discriminatory than measurement of function alone.

***Conclusions.*** Combined measurement of T-cell phenotype and function defines a highly discriminatory biomarker of tuberculosis disease activity. Unlocking the diagnostic and monitoring potential of this combined approach now requires validation in large-scale prospective studies.

The high proportion of the global tuberculosis burden among those with human immunodeficiency virus (HIV) coinfection results in poor individual and population level outcomes due to increased susceptibility, morbidity, and mortality [1]. *Mycobacterium tuberculosis* (M. tuberculosis) induces several conventional and unconventional T-cell subsets, but the predominant response is mediated by classically restricted, peptide-specific Th1 type CD4^+^ T cells and CD8^+^ cytotoxic T lymphocytes (CTLs), which are essential for protective immunity in murine models of tuberculosis [2]. HIV and active tuberculosis both impact M. tuberculosis-specific T-cell immunity, as evidenced by the phenomenon of skin test anergy and impaired cellular immunity in those with active tuberculosis without HIV coinfection [3] and HIV coinfected individuals [4]. Dissecting out the effects of tuberculosis stage and HIV infection is thus necessary to delineate the potential roles of distinct T-cell subsets as biomarkers of active tuberculosis and latent tuberculosis infection.

Functional CD4^+^ and CD8^+^ T-cell subsets have been defined based on single-cell cytokine (interferon γ [IFN-γ]/interleukin 2 [IL-2]/tumor necrosis factor α [TNF-α]) signatures. These are differentially impacted by disease stage, mycobacterial load, and treatment [5–7], suggesting that certain subsets may serve as biomarkers of disease activity, pathogen burden, or treatment response. However, evidence to date is limited by a paucity of data on the cell surface marker phenotype of these subsets in M. tuberculosis infection, which is key to characterization of T cells, denoting memory status, disease site homing, survival, and activation [8]. Changes in dominant functionally defined memory response have been associated with varying antigen load in other disease models [9, 10], and studies suggest that M. tuberculosis-specific cells present in active tuberculosis are predominantly of effector-memory phenotype [11–13]. However, further work is needed to confirm and further understand how memory and activation phenotype relates to tuberculosis disease stage [6] and HIV coinfection.

Previous data indicated that measurement of CD4^+^ M. tuberculosis-specific TNF-α-only secreting cells might serve as an accurate biomarker of active tuberculosis [14]. We hypothesized that measuring both M. tuberculosis-specific T-cell function and phenotype would refine this approach and reveal more discriminatory biomarker(s). Therefore, we performed multiparameter flow cytometry for 3 canonical cytokines and key markers of memory and activation in subjects distinguished by mycobacterial load (active tuberculosis vs latent tuberculosis infection) and HIV status. This enabled simultaneous definition of functional and phenotypic M. tuberculosis-specific T-cell profiles at the single-cell level. Studying precisely defined patient groups enabled us to dissect out the influence of mycobacterial load and HIV coinfection on M. tuberculosis-specific cellular immunity to tease out which functional and phenotypic subsets could serve as markers of mycobacterial pathogen burden independently of HIV coinfection status.

## METHODS

Participants were prospectively enrolled from 3 clinical centers in London, during the period January 2008–February 2011 under National Research Ethics Service approval (07/H0712/85). Participants were ≥18 years, provided written, informed consent, and were eligible if under clinical investigation for active tuberculosis, undergoing latent tuberculosis infection screening, or had recognized tuberculosis risk factors (eg, known tuberculosis contact).

Suspected active tuberculosis was confirmed microbiologically by the clinical diagnostic laboratory. Latent tuberculosis infection was defined as a positive response to RD-1 antigens in either T-SPOT.TB (carried out in routine clinical work up) or M. tuberculosis IFN-γ ELISpot (carried out for the current study) in the absence of symptomatic, microbiological, or radiological evidence of active tuberculosis.

Presence of HIV infection was confirmed by third or fourth generation sero-assay performed by the clinical diagnostic laboratory and using HIV-1 type specific enzyme immunoassay (EIA), according to national standards. HIV viral load (VL) and CD4 T-lymphocyte counts were assayed in the local Clinical Pathology Association-accredited diagnostic laboratories at the time of study recruitment. HIV diagnostics were available for all patients with active tuberculosis (in line with the national screening policy) and the majority of those with latent tuberculosis infection; the remainder had no risk factors for HIV and normal CD4:CD8 lymphocyte ratios and were classified as HIV-uninfected.

### IFN-γ M. tuberculosis ELISpot

Fresh or frozen peripheral blood mononuclear cells (PBMCs), 2.5 × 10^5^ per well, were stimulated overnight (37°C, 5% CO_2,_ 16–20 hours) in an IFN-γ ELISpot plate (Mabtech) with phytohemagglutinin (PHA; positive control; Sigma-Aldrich), Tuberculin Purified Protein Derivative (PPD; Statens Serum Institute) or pools of M. tuberculosis-specific 15-mer overlapping peptides covering each of ESAT-6, CFP-10, EspC, TB7.7, Rv3879c, Rv3873, and Rv3878. Unstimulated cells were used as a negative control. The IFN-γ ELISpot assay was performed as described elsewhere [15].

### Intracellular Cytokine Staining and Polychromatic Flow Cytometry

Thawed PBMCs (3–5 × 10^6^ per well) were cultured for 16 hours (37°C, 5% CO_2_) in 10% human serum (Sigma–Aldrich) in RPMI-1640 (Sigma-Aldrich) at a concentration of 1 × 10^7^ cells/mL. Cells were stimulated with PMA-Ionomycin (positive control) (Sigma–Aldrich; final concentration of 5ng/ml for PMA and 500ng/ml for Ionomycin), PPD (16.7 μg/mL final concentration), or a cocktail of peptides spanning the length of 3 highly immunodominant M. tuberculosis-specific RD1-associated antigens, ESAT-6, CFP-10, and EspC (10 µg/mL final concentration per peptide) [16]. Unstimulated cells were used as a negative control. After 2 hours, monensin (2 μM final concentration) was added. Following stimulation, cells were washed and stained with a dead cell marker (LIVE/DEAD Fixable Dead Cell Stain Kits, aqua, Invitrogen) for 30 minutes at 4°C in phosphate-buffered saline (PBS). Cells were then washed in PBS and placed in FC block buffer (10% human serum in filtered FACS solution [0.5% bovine serum albumin and 2 mM EDTA in PBS]) for 20 minutes at 4°C before staining with a pre-titrated and optimized antibody cocktail with fluorochrome-conjugated antibodies against CD3-APC-Alexa Fluor750, CD4-Qdot605, CD45RA-Qdot655 (Invitrogen), CD8-APC, CCR7-PE-Cy7, CD127-FITC (BD Biosciences), and PD-1-PerCP/Cy5.5 (Biolegend). After washing, the cells were fixed and permeabilized using BD Cytofix/Cytoperm Fixation/Permeabilization kit (BD Biosciences) for 20 minutes at 4°C. The cells were washed twice with Perm/Wash solution (BD Biosciences) then stained with pre-titrated fluorochrome-conjugated antibodies in Perm/Wash solution with IFN-γ-V450, IL-2-PE, and TNF-α-AlexaFluor 700 (BD Biosciences) for 30 minutes at 4°C. 1 × 10^6^ events (where possible) were acquired straightaway using a BD LSR-II flow cytometer. Anti-Rat and Anti-Mouse Ig compensation beads (BD Biosciences) were used to set compensation parameters. Fluorescence minus one (FMO) controls were used in each experiment to set gates.

### Data Analysis and Thresholds

The data were analyzed on FlowJo version 9.4.4,TreeStar, Inc. Events were gated on live cells, singlets, and lymphocytes using forward and side scatter properties. CD3^+^CD4^+^ and CD3^+^ CD8^+^ subsets were defined. Gating controls were used to define IFN-γ, IL-2, and TNF-α responses and surface marker expression.

For phenotypic analysis of M. tuberculosis-specific cells, only participants with a positive response were included. Positive responders were defined as those with a response that was ≥2 times the background (in unstimulated but fully stained samples) and >0.001% of CD3^+^CD4^+^ or CD3^+^CD8^+^ cells. This cutoff was used because we did not use costimulation to enhance responses, and we normalized to background (unstimulated) data before applying the cutoff (rather than classifying background as negligible). A strict cutoff meant only antigen-specific cells were included in the phenotypic analysis.

### Statistical Analysis

Statistical analysis was conducted using IBM SPSS Statistics version 20 and GraphPad Prism version 5.00 for Mac OS X, GraphPad Software, California. Tuberculosis disease stage compared all tuberculosis (n = 13) vs all latent tuberculosis infection (n = 21) regardless of HIV status, the impact of HIV compared all HIV-infected (n = 17) vs uninfected (n = 17) regardless of tuberculosis disease stage and across all 4 subgroups. The 2-tailed Mann–Whitney *U* test was used for nonparametric 2-sample comparisons. Spearman rank correlation coefficients were used to test correlations. Receiver operator characteristic (ROC) curve defined the sensitivity and specificity of the diagnostic approach.

## RESULTS

### Study Population

Demographic characteristics of the study population are shown in Table 1.

**Table 1.** Demographics and Clinical Test Results of Participants

	HIV/Tuberculosis		Tuberculosis		HIV/Latent Tuberculosis Infection		Latent Tuberculosis Infection		Total	
	7	(%)	6	(%)	10	(%)	11	(%)	34	(%)
Median (IQR) age	43	(40.5–52.4)	34.5	(28.0–56.0)	36	(24.0–39.0)	33	(31.0–35.5)	35.5	(31.3–40.8)
Sex										
Male	4	(57.1)	3	(50.0)	6	(60.0)	4	(36.4)	17	(50.0)
Female	3	(42.9)	3	(50.0)	4	(40.0)	7	(63.6)	17	(50.0)
Ethnicity										
Black	5	(71.4)	1	(16.7)	8	(80.0)	6	(54.5)	20	(58.8)
Asian	1	(14.3)	3	(50.0)	0	(0.0)	3	(27.3)	7	(20.6)
White	1	(14.3)	2	(33.3)	2	(20.0)	2	(18.2)	7	(20.6)
BCG vaccination										
yes	5	(71.4)	3	(50.0)	9	(90.0)	8	(72.7)	25	(73.5)
no	1	(14.3)	2	(33.3)	0	(0.0)	2	(18.2)	5	(14.7)
unknown	1	(14.3)	1	(16.7)	1	(10.0)	1	(9.1)	4	(11.8)
Microbiological (smear/culture) confirmation										
positive	7^a^	(100.0)	6^a^	(100.0)	NA	NA	NA	NA	13	(100.0)
negative	0	(0.0)	0	(0.0)	NA	NA	NA	NA	0	(0.0)
HIV test										
positive	7	(100.0)	0	(0.0)	10	(100.0)	0	(0.0)	17	(50.0)
negative	0	(0.0)	6	(100.0)	0	(0.0)	6	(54.5)	12	(35.3)
not done	0	(0.0)	0	(0.0)	0	(0.0)	5^b^	(45.5)	5	(14.7)

Abbreviations: HIV, human immunodeficiency virus; IQR, interquartile range; NA, not applicable.

All subjects tested positive in one or more of the tuberculin skin test, TSPOT.*TB*, QuantiFERON-TB Gold In-Tube or M. tuberculosis IFN-γ ELISpot, performed clinically or for the current study.

^a^ 10 patients with tuberculosis had not started treatment at the point of recruitment, 2 had received <14 days treatment, and 1 had received ≥14 days treatment.

^b^ All participants without clinical need for HIV testing had normal CD4:CD8 ratios.

### The Frequency of CD4^+^ and CD8^+^ Cells With an IFN-γ^+^ and TNF-α^+^ Response Was Increased in Active Tuberculosis

**Figure 1.**
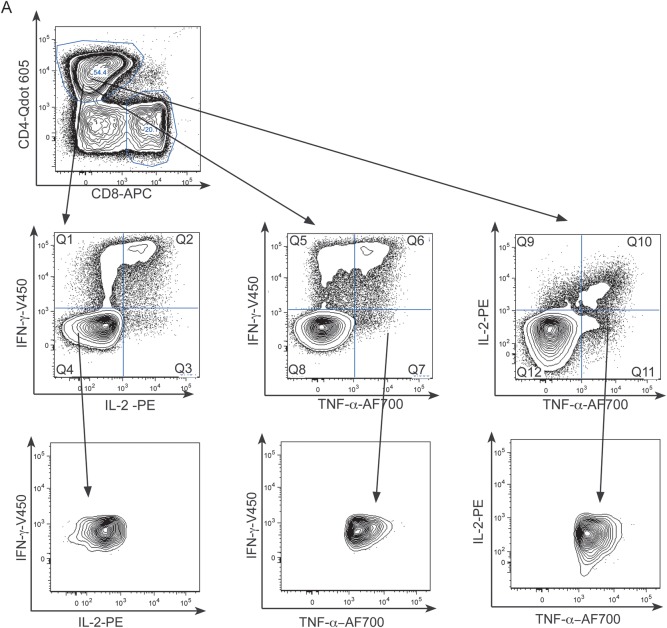

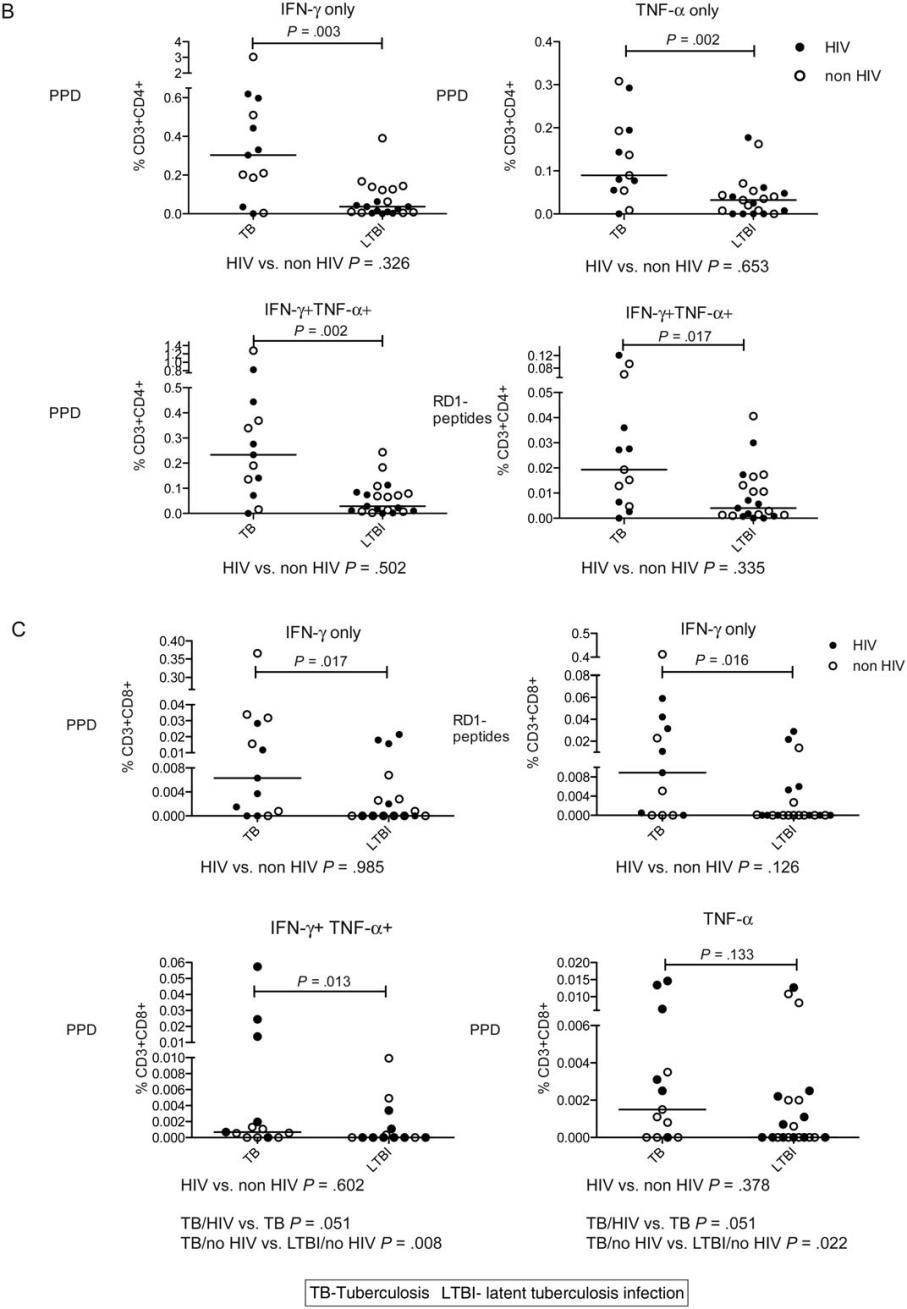
Frequency of IFN-γ and TNF-α secreting CD4^+^ and CD8^+^ cell subsets are increased in active tuberculosis. *A*, Example gating strategy for the CD4^+^ TNF-α-only-secreting subset using representative plots from an individual with active tuberculosis whose cells were stimulated overnight with PPD is shown. Cells were gated on live singlets (not shown) and CD3 ^+^ CD4^+^ cells (*top row*), then according to IFN-γ, IL-2, and TNF-α expression using FMOs (*middle row*). Boolean gating was used to define individual nonoverlapping functional subsets, eg, the TNF-α-only subset which did not express IFN-γ or IL-2 (*bottom row*). Graphs show frequency and median of CD4^+^ (*B*) and CD8^+^ (*C*) cells secreting IFN-γ and TNF-α in response to overnight stimulation with PPD or RD1-peptides in participants with active tuberculosis vs latent tuberculosis infection. Those with HIV coinfection (*filled circles*) and without HIV coinfection (*open circles*) are indicated. Results were analyzed by Mann–Whitney *U* test; and *P* values of < .05 were considered significant. Abbreviations: HIV, human immunodeficiency virus; IFN, interferon; IL, interleukin; PPD, purified protein derivative; TNF, tumor necrosis factor.

We first examined the frequency of CD4^+^ and CD8^+^ functional effector cell subsets. Boolean gating was used to create individual nonoverlapping subsets by combining data in 3 dimensions (Figure 1*A*). The frequency of PPD-specific CD4^+^ IFN-γ only, TNF-α only, and IFN-γ/TNF-α-dual-secreting cells was higher in active tuberculosis compared with latent tuberculosis infection (*P* = .003, .002, and .002, respectively; Figure 1*B*). A similar relationship was seen for RD1-peptide-specifc CD4^+^ IFN-γ-only-secreting cells (not significant; data not shown), and IFN-γ/TNF-α-dual-secreting cells (*P* = .017; Figure 1*B*). The presence of HIV infection was not associated with an altered frequency of these cell subsets. We observed no difference in the frequency of trifunctional cells in patients with active tuberculosis compared with those with latent tuberculosis infection (Supplementary Figure 1).

The majority of participants with active tuberculosis, but not latent tuberculosis infection, had a CD8^+^ IFN-γ response (PPD: 12/13 for active tuberculosis vs 6/21 for latent tuberculosis infection, RD1-peptides: 10/13 for active tuberculosis vs 11/21 for latent tuberculosis infection). The frequencies of PPD- and RD1-peptide-specific CD8^+^ IFN-γ-only producing cells were significantly higher in active tuberculosis than in latent tuberculosis infection (*P* = .017 and .016, respectively), as was the frequency of CD8^+^ PPD-specific cells secreting both IFN-γ and TNF-α (*P* = .013; Figure 1*C*). HIV coinfection was (nonsignificantly) associated with a reduced frequency of PPD-specific IFN-γ/TNF-α-dual and TNF-α-only responses in active tuberculosis compared with HIV negativity (*P* = .051 for both). In the HIV-uninfected tuberculosis group the percentages of these PPD-specific CD8^+^ cells were significantly higher than in latent tuberculosis infection (*P* = .008 and .022, respectively). Similarly, the frequency of cells secreting IFN-γ-only were significantly higher in active tuberculosis compared with latent tuberculosis infection in HIV-uninfected (*P* = .023). These CD8^+^ effector functional subsets were therefore related to mycobacterial load analogously to equivalent CD4^+^ subsets, but the impact of HIV coinfection was more profound.

### PPD-specific and RD1-peptide-specific CD4^+^ Cellular Differentiation Was Increased in Active Tuberculosis vs Latent Tuberculosis Infection

**Figure 2.**
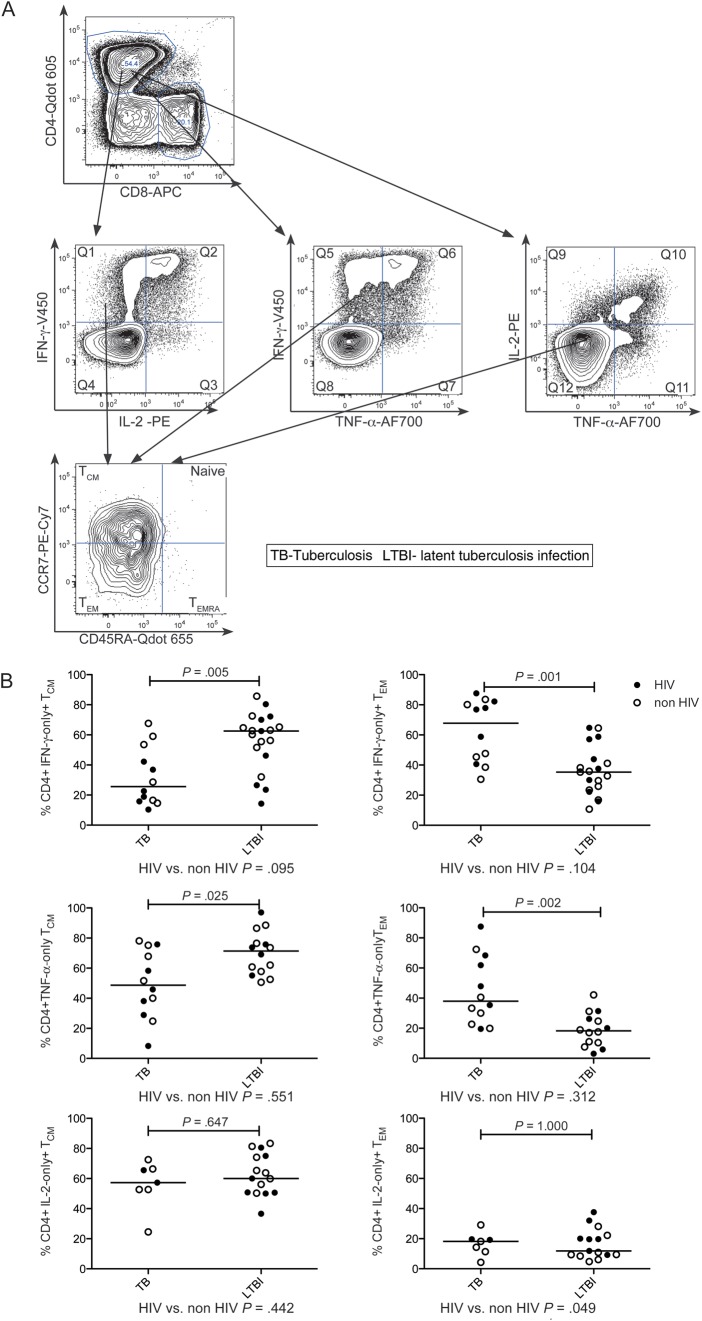
Cell surface phenotype of CD4^+^ cell functional subsets is influenced by tuberculosis disease stage: CD4^+^ cell functional subsets were examined for CD45RA and CCR7 expression in active vs latent tuberculosis infection in those with a positive response. *A*, An example gating strategy for a PPD-specific CD4^+^ IFN-γ-only secreting subset is demonstrated using representative plots from an individual with active tuberculosis. Each CD3 ^+^ CD4^+^ (*top row*) functional subset, eg, IFN-γ-only-secreting cells (*middle row*) was analyzed for expression of CD45RA and CCR7 (*bottom row*). *B*, Graphs show the percentage (and median percentage) of PPD-stimulated CD4^+^ IFN-γ-only (*top row*), TNF-α-only (*middle row*), and IL-2-only (*bottom row*) cells that were CD45RA-CCR7^+^ (T_CM_) (*left column*) and CD45RA-CCR7- (T_EM_) (*right column*) in patients with active tuberculosis and latent tuberculosis infection. The top 2 rows are representative of changes observed in active vs latent tuberculosis infection in all M. tuberculosis-specific (responding to PPD and RD-1-peptides) CD4^+^ functional subsets except IL-2-only-secreting cells. Those with HIV coinfection (*filled circles*) and without HIV coinfection (*open circles*) are indicated. Results were analyzed by Mann Whitney *U* test; and *P* values of < .05 were considered significant. Abbreviations: HIV, human immunodeficiency virus; IFN, interferon; IL, interleukin; M. tuberculosis, *Mycobacterium tuberculosis*; PPD, purified protein derivative; TNF, tumor necrosis factor.

We analyzed the memory phenotype of the CD4^+^ functional subsets as putative correlates of mycobacterial pathogen load. Nonresponders were excluded. Each T-cell functional subset was gated for expression of CD45RA and CCR7 (Figure 2*A*). Memory phenotypes of functional subsets were defined as naive CD45RA ^+^ CCR7^+^, central memory (T_CM_) CD45RA^−^ CCR7^+^, effector memory (T_EM_) CD45RA^−^CCR7^−^ and CD45RA^+^ effectors (T_EMRA_) CD45RA^+^ CCR7^−^. This last subset was mainly evident in CD8^+^ cells.

PPD- and RD1-peptide-specific CD4^+^ cell effector functional subsets were principally T_CM_ in latent tuberculosis infection compared to T_EM_ in active tuberculosis, for example, fewer PPD-specific CD4^+^ IFN-γ-only secreting cells were T_CM_ in active tuberculosis compared with latent tuberculosis infection (*P* = .005; Figure 2*B*). Comparisons of HIV-infected with uninfected patients were nonsignificant except for the CD4^+^ RD1-peptide-specific IFN-γ-only- and TNF-α/IL-2-dual-secreting subsets, fewer of which were T_CM_ in HIV-infected than uninfected subjects (*P* = .030 and .006; Supplementary Figure 2).

In contrast to the CD4^+^ effector functional subsets, CD4^+^ IL-2-only PPD- (Figure 2*B*) and RD1-peptide-specific cells (data not shown) were principally T_CM_ in both active tuberculosis and latent tuberculosis infection and were therefore unaffected by tuberculosis disease stage. CD8^+^ IFN-γ-only cellular responses to PPD and RD1-peptides were mostly T_EM_ and T_EMRA_ (data not shown).

**Figure 3.**
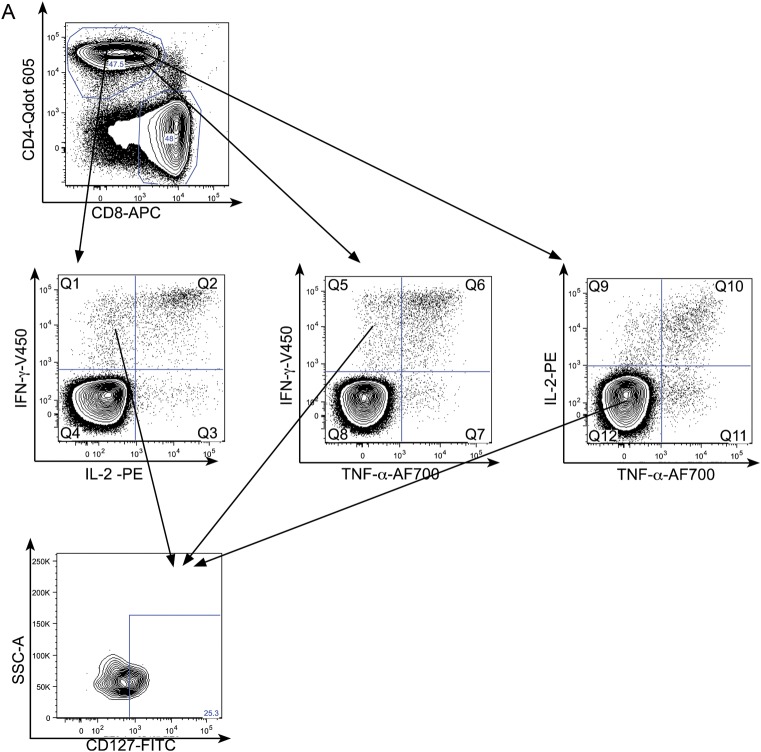

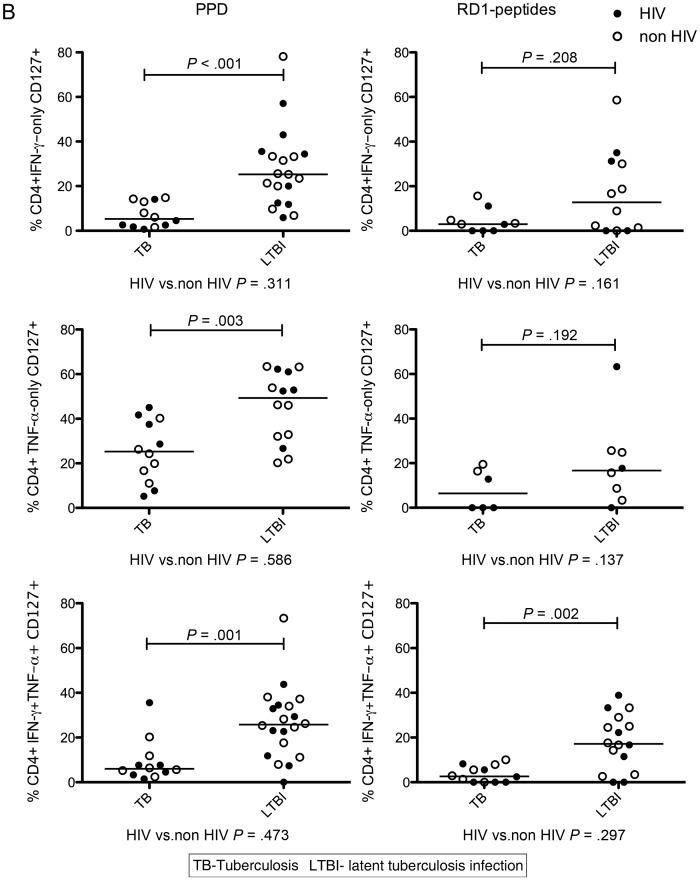

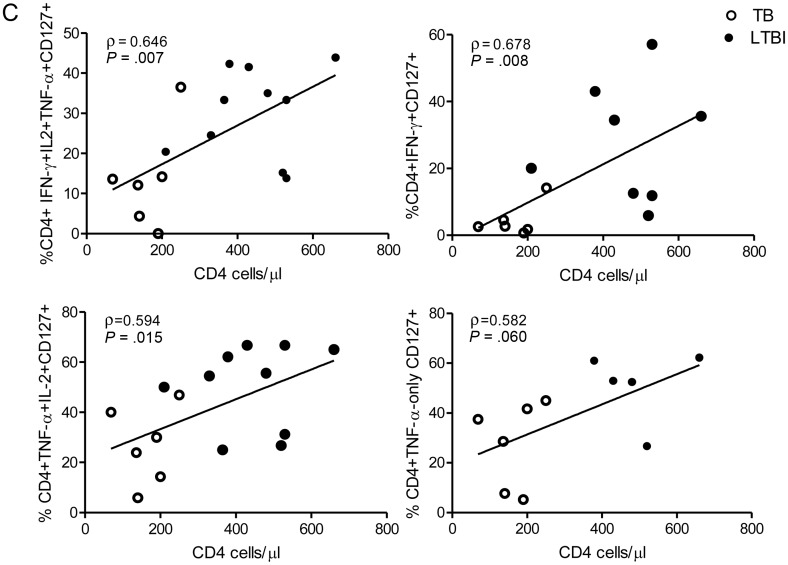
Percentage of M. tuberculosis-specific CD4^+^ functional T-cell subsets expressing CD127 is influenced by stage of tuberculosis infection and CD4 count: CD3 ^+^ CD4^+^ functional cell subsets were examined for CD127 expression. *A*, A representative gating strategy is shown. PBMCs from an individual with latent tuberculosis infection infection were stimulated overnight with PPD, and CD3 ^+^ CD4^+^ cells (*top row*) were gated for cytokine secretion, eg, IFN-γ-only-secreting subset (*middle row*) and analyzed for expression of CD127 (*bottom row*).

CD127 (IL7Rα) expression is reduced following antigen stimulation in effector T cells [17] and defines CD4^+^ and CD8^+^ subsets of differentiated murine T effector cells distinct from effector memory cells [18, 19]. We therefore measured CD127 expression on antigen-specific CD4^+^ functional T-cell subsets (Figure 3*A*). A smaller percentage of PPD- and RD1-peptide-specific CD4^+^ cells expressed CD127 in active tuberculosis compared with latent tuberculosis infection, for example, a lower percentage of PPD-specific CD4^+^ IFN-γ-only- and TNF-α-only-secreting cells expressed CD127 in active tuberculosis compared with latent tuberculosis infection (*P* < .001 and *P* = .003, respectively; Figure 3*B*). Expression of CD127 on antigen-specific cells was unaffected by HIV status (Figure 3*B*). However, in patients with HIV coinfection, frequencies of several subsets of PPD-specific T cells expressing CD127 correlated with CD4 count (Figure 3*C*). Similarly, for RD-1-peptide-specific cells, IFN-γ/IL-2-dual-producing cells expressing CD127 correlated with CD4 count (ρ = 0.647; *P* = .047) and HIV VL correlated inversely with IFN-γ-only (ρ = −0.780; *P* = .013) and IFN-γ/TNF-α-dual-secreting CD127-expressing cells (ρ = −0.727; *P* = .005; data not shown).

### Expansion of Differentiated CD4^+^ Functional Effector T Cells in Active Tuberculosis vs Latent Tuberculosis Infection

**Figure 4.**
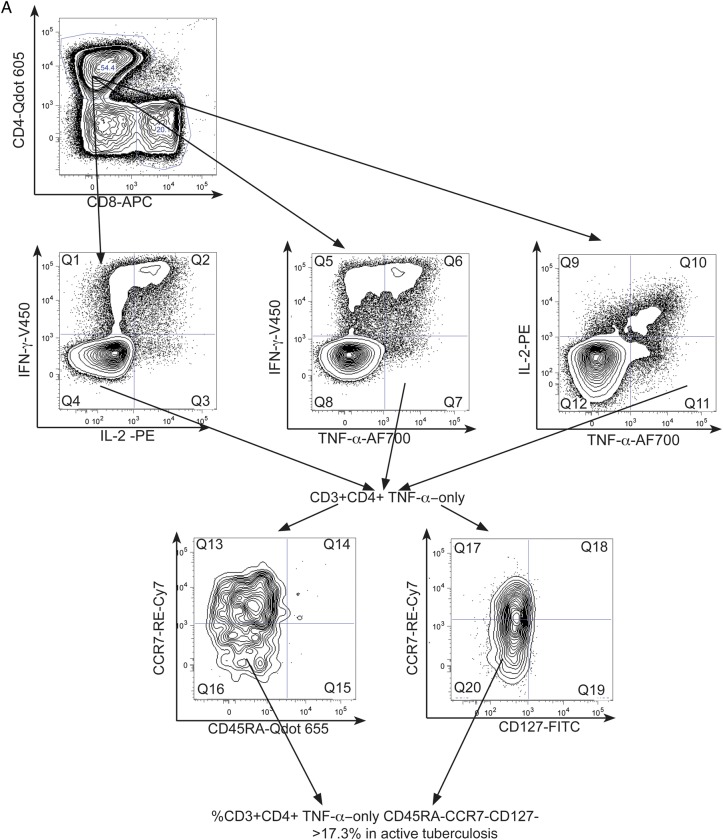

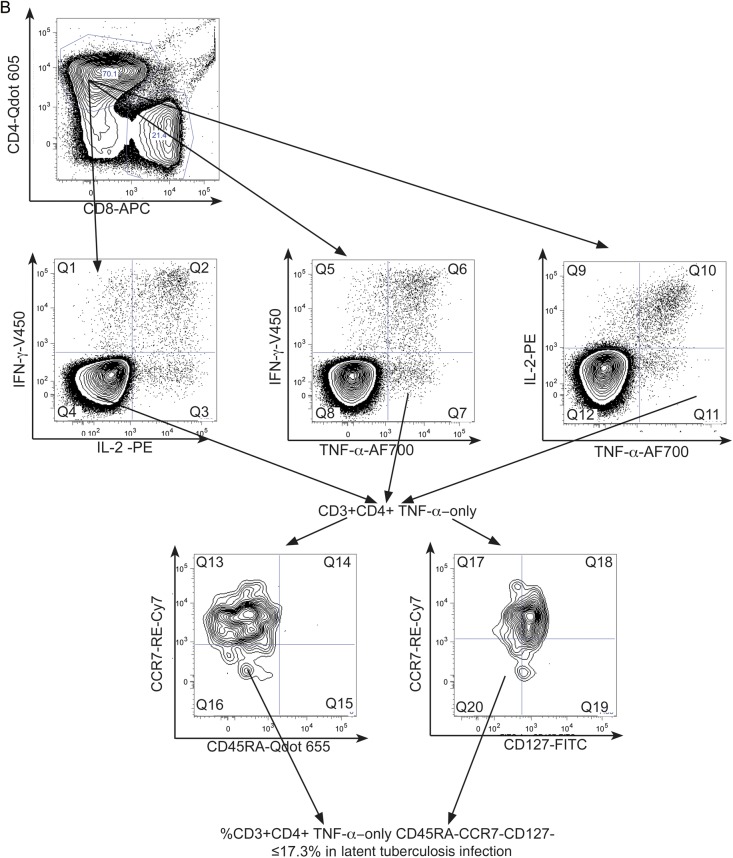

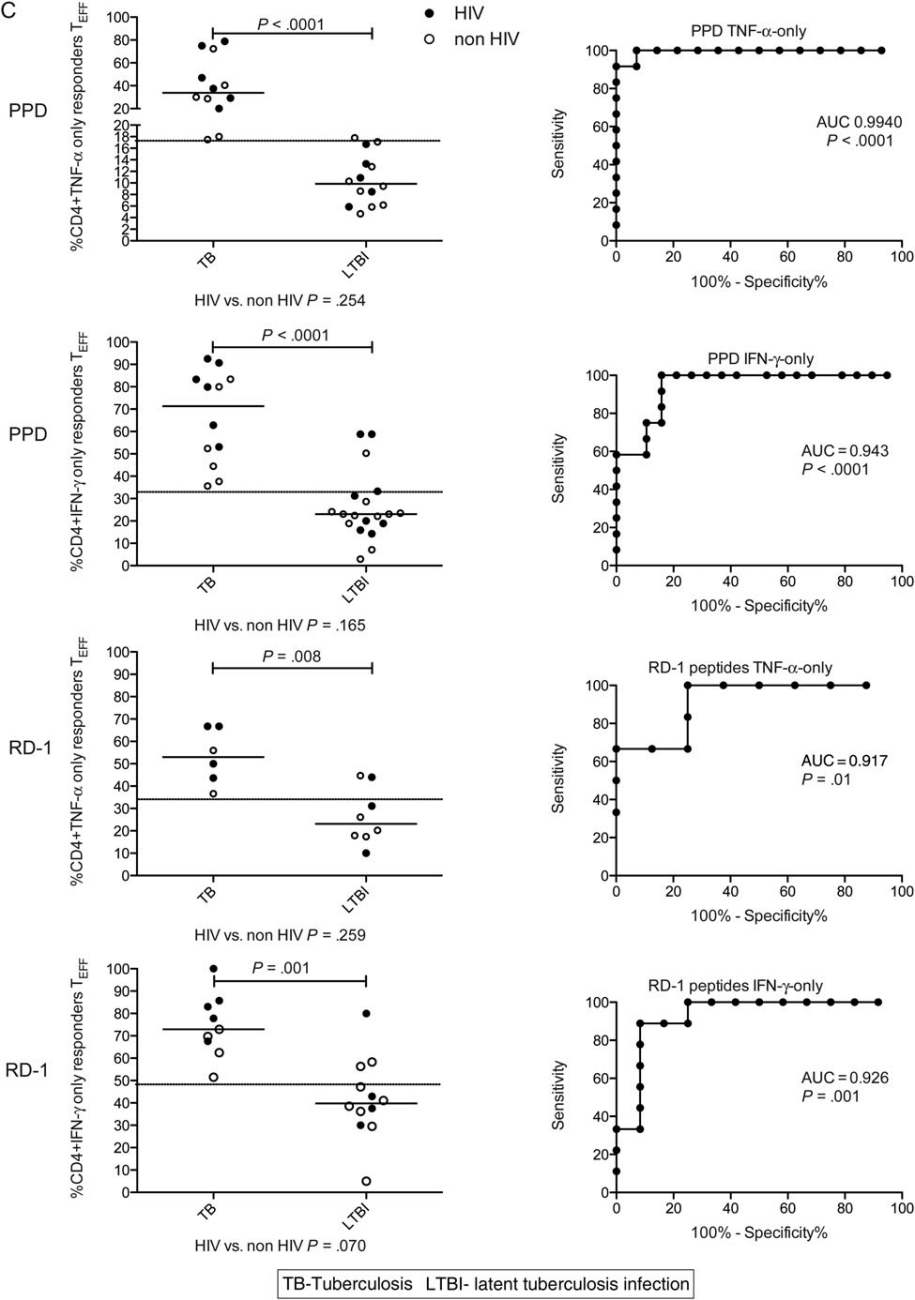

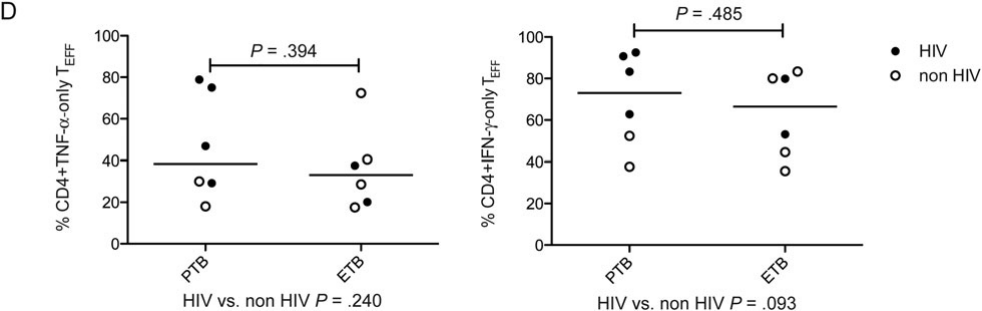
Combining functional subset analysis with memory phenotype reveals a potentially powerful biomarker to distinguish active and latent tuberculosis infection. Boolean gating was used to analyze the percentage of PPD-specific CD4^+^ TNF-α-only-secreting cells that had the phenotype T_EFF_ (CD45RA-CCR7-CD127-) in active and latent tuberculosis infection. A representative gating strategy is shown for individuals with active tuberculosis (*A*) and latent tuberculosis infection (*B*).

We next investigated the potential of combined phenotypic and functional measurement as a clinical biomarker. We determined the percentage of PPD- and RD1-peptide-specific CD4^+^ cells secreting IFN-γ-only or TNF-α-only that were differentiated effector cells (T_EFF_; CD45RA-CCR7-CD127-; Figure 4*A* [active tuberculosis] and B [latent tuberculosis infection]). In active tuberculosis, compared with latent tuberculosis infection, a higher percentage of CD4^+^ cells secreting IFN-γ-only or TNF-α-only in response to PPD and RD1-peptides were T_EFF_. This was most significant for CD4^+^ PPD-specific CD4^+^ cells secreting TNF-α-only (*P* < .0001) and IFN-γ-only (*P* < .0001). A cutoff of 17.3% of TNF-α-only cells of T_EFF_ phenotype distinguished active tuberculosis from latent tuberculosis infection with 100% sensitivity (95% CI, 73.5–100.0) and 92.9% specificity (95% CI, 66.1–99.8; Table 2). In ROC analysis, area under the curve was 0.99 (95% CI, .97–1.01; *P* < .0001; Figure 4*C*). Similar, although slightly less discriminatory ROC curves were generated for PPD-specific IFN-γ-only cells and for RD-1-specific cells (Figure 4*C*).

**Table 2.** Clinical and Radiological Characteristics of Cases Sorted by Percentage of TNF-α-only-Secreting Cells That Were T_EFF_ (CD45RA^−^CCR7^−^CD127^−^)

No.	%T_EFF_/TNF-α only	HIV	CD4	VL	Sputum smear	M. tuberculosis culture	Culture site	Radiology (CXR or CT)	Tuberculosis final diagnosis
S135	78.9	Pos	190	281 671	Neg	Pos	BAL and pleural fluid	Bilateral pleural effusions, lung and splenic nodules, peritoneal thickening	Pulmonary
S126	75.0	Pos	69	601 000	Pos	Pos	Sputum	Azygos lobe focal consolidation in cavity, pleural effusions, no lymphadenopathy	Pulmonary
S221	72.4	Neg	NA	NA	NT	Pos	Lymph node	Enlarged low density lymph nodes in mediastinum and left axilla	Extra pulmonary
S059	46.9	Pos	140	<100	Pos	Pos	Sputum and BAL	Effusion, thickened pleura, loss of volume left lung, ground glass change	Pulmonary
S184	40.5	Neg	NA	NA	NT	Pos	Lymph node	Multiple mediastinal, coeliac axis lymph nodes with nodules in spleen and breast	Extra pulmonary
S193	37.6	Pos	136	28 958	NT	Pos	Lymph node	Axillary, para-aortic, and abdominal lymphadenopathy, subpleural nodules, liver lesions	Extra pulmonary
S083	30.0	Neg	NA	NA	Neg	Pos	Lymph node, BAL, peritoneal	Right pleural collection and right paratracheal lymphadenopathy	Pulmonary
S076	29.2	Pos	200	<50	Pos	Pos	Left upper lobe, BAL, sputum	Consolidation and cavitation upper lobe, interstitial opacities, linear atelectasis	Pulmonary
S115	28.6	Neg	NA	NA	NT	Pos	Lymph node	Mediastianal lymphadenopathy	Extra pulmonary
S146	20.1	Pos	250	52 205	NT	Pos	Lymph node	Supraclavicular, mediastinal, and abdominal lymphadenopathy, nodular infiltrates	Extra pulmonary
S195	18.0	Neg	NA	NA	Pos	Pos	BAL and lymph node	Mediastinal, hilar, and supraclavicular lymph nodes, patchy consolidation	Pulmonary
S153	17.8	Neg	NA	NA	NT	NT	NT	Nil of note	Latent tuberculosis infection
S082	17.5	Neg	NA	NA	Neg	Pos	Lymph node	Nil of note	Extra pulmonary
S074	17.1	Neg	NA	NA	Neg	*M fortuitum*	BAL	Opacification right upper lobe, small volume axillary, and mediastinal lymph nodes	Latent tuberculosis infection
S050	16.7	Pos	480	12 479	NT	NT	NT	Nil of note	Latent tuberculosis infection
S052	13.3	Pos	520	45 719	Neg	Neg	Sputum	Nil of note	Latent tuberculosis infection
S177	12.8	Neg	NA	Na	NT	NT	NT	Nil of note	Latent tuberculosis infection
S094	10.9	Pos	660	<50	NT	NT	NT	Heavily calcified nodule and small lymph nodes right upper lobe	Latent tuberculosis infection
S092	10.3	NT	NA	NA	NT	NT	NT	Nil of note	Latent tuberculosis infection
S145	9.4	Neg	NA	NA	NT	NT	NT	NT	Latent tuberculosis infection
S098	8.6	Neg	NA	NA	NT	NT	NT	(Fractured ribs T4–9 posteriorly)	Latent tuberculosis infection
S047	8.5	Pos	360	<50	NT	NT	NT	Nil of note	Latent tuberculosis infection
S079	6.2	NT	NA	NA	NT	NT	NT	Nil of note	Latent tuberculosis infection
S001	5.9	Pos	430	775	NT	NT	NT	Nil of note	Latent tuberculosis infection
S099	5.8	NT	NA	NA	NT	NT	NT	Nil of note	Latent tuberculosis infection
S120	4.7	NT	NA	NA	NT	NT	NT	Fibrosis both apices, hilar lymphadenopathy, pleural thickening	Latent tuberculosis infection
S097	NA	Pos	177	19 906	Pos	Neg	Sputum	Subcarinal and axillary lymph nodes	Pulmonary
S029	NA	Pos	530	<50	NT	NT	NT	Nil of note	Latent tuberculosis infection
S025	NA	Pos	330	<50	NT	NT	NT	Nil of note	Latent tuberculosis infection
S197	NA	Pos	210	<50	NT	NT	NT	Nil of note	Latent tuberculosis infection
S201	NA	Neg	NA	NA	NT	NT	NT	Nil of note	Latent tuberculosis infection
S171	NA	Pos	365	<50	NT	NT	NT	Nil of note	Latent tuberculosis infection
S191	NA	Pos	530	13 328	NT	NT	NT	NT	Latent tuberculosis infection
S121	NA	Neg	NA	NA	NT	NT	NT	Nil of note	Latent tuberculosis infection

Abbreviations: BAL, bronchoalveolar lavage; CT, computerized tomography; CXR, chest x-ray; HIV, human immunodeficiency virus; NA, not applicable; Neg, negative; NT, not tested; Pos, positive; TNF, tumor necrosis factor; VL, viral load.

Those with >17.3% TEFF/TNF-alpha only cells would be predicted to have active tuberculosis in the original analysis and those below it to have latent tuberculosis infection. Those below the second bold dividing line did not have a positive TNF-α-only response to purified protein derivative.

To test whether this approach was robust to differences in disease site, we compared individuals with active pulmonary and extrapulmonary disease. We found no significant difference in the proportion of PPD-specific cells secreting IFN-γ-only, or TNF-α-only that were T_EFF_ (Figure 4*D*) when stratified by site of disease or HIV coinfection. There was also no difference in the proportion of RD-1 peptide-specific cells that were T_EFF_ when stratified by disease site (data not shown).

The operator conducting analyses (KMP) was also involved in participant recruitment and therefore not blinded to patient categorization. To demonstrate integrity and reproducibility of the results, data for all study participants were regated and reanalysed by a second independent operator (HSW) who was blinded to patient diagnoses. Correlations between results obtained by operators 1 and 2 for the percentage of CD3 ^+^ CD4^+^ cells secreting TNF-α-only (ρ = 0.97, *P* < .0001), the percentage of CD3 ^+^ CD4^+^ TNF-α-only-secreting cells that are CD127^+^ (ρ = 0.96, *P* < .0001) and the percentage of CD3 ^+^ CD4^+^ TNF-α-only-secreting cells that are CD45RA^−^CCR7^−^ (ρ = 0.88, *P* < .0001) were very strong. Using the 17.3% cutoff for TNF-α-only-secreting cells of T_EFF_ phenotype to distinguish tuberculosis from latent tuberculosis infection, operator 2 misclassified 1 case of tuberculosis as latent tuberculosis infection, and 1 further case of latent tuberculosis infection as tuberculosis (data not shown).

## DISCUSSION

Our detailed interrogation of antigen-specific T-cell phenotype and function has delineated the association of tuberculosis disease stage with M. tuberculosis-specific cellular immunity. active tuberculosis was associated with an increased frequency of mono- or dual-functional CD4^+^ and CD8^+^ M. tuberculosis-specific T cells that secrete IFN-γ and/or TNF-α making these subsets potential biomarkers of disease activity.

Measurement of the proportion of TNF-α-only secreting M. tuberculosis-responsive CD4^+^ cells has proved promising to distinguish between active tuberculosis and latent tuberculosis infection [14] but neither earlier data nor our findings have clinically sufficient discriminatory power. The slight difference in our findings compared with those previously published may be because we stratified for HIV infection and tuberculosis disease stage and we did not use costimulation in our assays. The use of costimulation may reflect the cells’ ability to react to certain stimuli not necessarily present in vivo, instead of measuring the ability of these cells to respond to antigen. Studies have suggested that the presence of trifunctional cells secreting IFN-γ, IL-2, and TNF-α correlates with active tuberculosis [20, 21], but our data did not support this association. Our functional data therefore partially corroborates and extends earlier observations [6, 14] that measurement of the frequency of M. tuberculosis-specific CD4^+^ effector-like cells secreting IFN-γ and/or TNF-α can distinguish active tuberculosis from latent tuberculosis infection.

Simultaneous evaluation of memory phenotype of responding cells provided a more sensitive and specific surrogate than CD4^+^ functional profile alone. Expression of CD45RA, CCR7, and CD127 on M. tuberculosis-specific T cells secreting only IFN-γ or TNF-α was lowest in those with active tuberculosis. Interestingly, memory phenotype was not exclusively linked to the functional profile (except for IL-2-only cells which were mainly T_CM_) but was closely related to underlying tuberculosis stage. These markers might therefore serve as indicators of tuberculosis activation. Combined measurement of both functional profile and differentiation phenotype provided a highly discriminatory immunological read-out for active tuberculosis and latent tuberculosis infection. In those with active tuberculosis, >17.3% of PPD-specific CD4^+^ TNF-α-only-secreting cells were CD45RA^−^CCR7^−^CD127^−^, and this phenotype was therefore strongly associated with activated infection. Given that responses to PPD are less specific for M. tuberculosis infection than responses to RD-1 antigens, this PPD-specific T-cell signature may be used to distinguish tuberculosis from latent tuberculosis infection in the second step of a 2-step diagnostic testing strategy, where M. tuberculosis infection has been ruled in at step one by a positive result in an RD-1-based immunodiagnostic test (eg, IGRA).

Memory phenotype of responding cells and association with tuberculosis stage has previously been evaluated qualitatively and to some extent quantitatively. Studies have suggested that changes in the predominant memory phenotype of the responding CD4^+^ and CD8^+^ cells might be dependent on severe vs nonsevere, or active vs latent tuberculosis in adults and children [22–25]. None were able to simultaneously measure the antigen-specific function and phenotype of multiple CD4^+^ and CD8^+^ T-cell subsets. Equally these studies did not specifically measure cells with the T_EFF_ phenotype, which had lost CD127 expression. Therefore, although the principle was explored, none identified a subset that could serve as a highly sensitive and specific clinical biomarker.

The frequency of both PPD and M. tuberculosis-specific CD8^+^ functional effectors increased in active tuberculosis and might therefore also be responding to increased mycobacterial load, consistent with the observation that these cells respond more effectively to heavily infected dendritic cells [26]. The frequency of M. tuberculosis-specific CD8^+^ cells was increased in adults with sputum smear-positive pulmonary tuberculosis [6] and in children with active tuberculosis compared with contacts [27]. Similarly our data showed that the frequency of CD8^+^ M. tuberculosis-specific cells, and therefore proportion of responders, was increased in active tuberculosis and this approach holds promise for the discrimination of tuberculosis disease stage especially in HIV coinfection where all participants had a positive response to M. tuberculosis peptides. This association, shown by us and others [6], precludes the comparison of combined function and phenotype of M. tuberculosis-specific CD8^+^ cells, however, because nonresponders were by default mainly in the latent tuberculosis infection group. Measurement of CD8^+^ functional subsets in active tuberculosis and latent tuberculosis infection was therefore not sufficiently discriminatory for active and latent tuberculosis infection in our cohort.

In this study we included individuals with HIV coinfection to compare and distinguish the impact of active tuberculosis on M. tuberculosis-specific cellular immunity with the impact of HIV coinfection. Where CD4^+^ M. tuberculosis-specific effector-like cells were influenced by tuberculosis disease stage, the impact of HIV coinfection per se was rarely significantly associated with these changes. This may have been partially due to the inclusion of patients who were treated for HIV infection. However, in the case of CD127, stratification by CD4 count showed that the stage of HIV disease influenced expression of this marker on M. tuberculosis-specific T cells. Reduced CD127 expression on HIV-specific CD8^+^ T cells (reviewed in [28]) and CD4^+^ T cells [29] is observed with HIV disease progression, but a relationship between HIV disease progression and CD127 expression on M. tuberculosis-specific T cells has not previously been noted. Our finding indicates that in HIV coinfection, M. tuberculosis-antigen-specific CD4^+^ cells lose CD127 expression with advancing HIV disease and are therefore potentially more differentiated. This effect could be directly virus-induced or secondary to increasing subclinical mycobacterial burden with advancing HIV infection.

Our cohort included individuals with both pulmonary and extra-pulmonary infection. HIV infection is more commonly associated with tuberculosis dissemination as evidenced by the widespread involvement in some of these individuals. Despite some variation in clinical phenotype of those with active tuberculosis, our biomarker reliably distinguished tuberculosis stage, regardless of site of disease suggesting that it may remain robust across the clinical tuberculosis disease spectrum and therefore have wide applicability. HIV coinfection has recently been shown to be associated with subclinical active tuberculosis infection in a tuberculosis endemic area [30]. No individuals with latent tuberculosis infection developed active tuberculosis during 12 months of follow-up suggesting that, in our cohort (recruited in a tuberculosis nonendemic area), subclinical tuberculosis was not present in those classified as latent tuberculosis infection. The lack of continuous exogenous priming or restimulation due to tuberculosis exposure distinguishes our cohort from others recruited from tuberculosis endemic areas and removes this is as a possible confounding effect on the immunological changes we observed.

It is unknown whether the changes in function and phenotype we demonstrated are consequent upon, or causal of, increased disease activity. In a study of tuberculosis contacts there was no percentage difference in CD4^+^ or CD8^+^ memory phenotype at baseline in progressors and nonprogressors [31] arguing against a causal role. Rather, mycobacterial burden might be driving the dominant functional and phenotypic changes, strengthening the potential of these markers to serve as a surrogate of mycobacterial burden. The loss of CCR7, allowing T-cell accumulation at the site of disease [32], and the loss of CD127, limiting the number and life span of these potentially tissue-destructive cells, may reflect an active adaptive response to replicating mycobacteria. However, given that immune containment has, by definition, been lost in active tuberculosis, it is equally possible that these T-cell subsets contribute to tissue pathology, cavitation, and bacillary dissemination.

Limitations of this study included the modest cohort size and some heterogeneity. Our cohort was sufficient to reveal powerful positive associations with tuberculosis stage; but a larger study would be required to confirm the absence of associations, for example, between HIV coinfection and frequencies of certain T-cell subsets. Moreover, because most of our HIV-infected participants had relatively high CD4 cell counts, our findings may be less applicable to more immunosuppressed HIV-infected patients. Similarly, while our data suggested that our biomarker was independent of tuberculosis disease site (ie, pulmonary vs extrapulmonary tuberculosis), a larger sample size is needed to corroborate this. Such studies could include other antigens, for example, heparin-binding hemagglutinin adhesin, that have shown promise in distinguishing active tuberculosis from latent tuberculosis infection and risk stratification of those with infection [33, 34]

Through dissection of the impact of varying tuberculosis stage as a surrogate for mycobacterial pathogen burden, with and without HIV coinfection, we have identified cellular changes that are highly sensitive to tuberculosis activity. Future work will require prospective evaluation of our findings in an independent validation cohort, which if corroborative will have significant clinical impact.

## Supplementary Data

Supplementary materials are available at *The Journal of Infectious Diseases* online (http://jid.oxfordjournals.org/). Supplementary materials consist of data provided by the author that are published to benefit the reader. The posted materials are not copyedited. The contents of all supplementary data are the sole responsibility of the authors. Questions or messages regarding errors should be addressed to the author.

Supplementary Data
