# Supplementary material for: T-Cell Immunophenotyping Distinguishes Active From Latent Tuberculosis
Source: J Infect Dis. 2013 Sep 15;208(6):952–68. doi: 10.1093/infdis/jit265 (PMC3749005; doi:10.1093/infdis/jit265)
Supplement: Supplementary Data [file supp_208_6_952__index.html]

T-Cell Immunophenotyping Distinguishes Active From Latent Tuberculosis — Supplementary Data 

# T-Cell Immunophenotyping Distinguishes Active From Latent Tuberculosis

## 

Supplementary Data

**Files in this Data Supplement:**

- Supplementary Data - Docx file
- Supplementary Figure 1 - tif file
- Supplementary Figure 2 - tif file
