# Supplementary material for: T-Cell Immunophenotyping Distinguishes Active From Latent Tuberculosis
Source: J Infect Dis. 2013 Sep 15;208(6):952–68. doi: 10.1093/infdis/jit265 (PMC3749005; doi:10.1093/infdis/jit265)
Supplement: Supplementary Data [file supp_jit265_jit265supp.docx]

**Supplementary Figure Legends**

**Supplementary figure 1**

There was no difference in the frequency of tri-functional cells between those with ATB or LTBI or HIV-infected versus uninfected responding to PPD (top row) or RD-1 peptides (bottom row). Patients with HIV co-infection (filled circles) and without HIV co-infection (open circles) are indicated. Results were analysed by Mann Whitney U test; and p values of <0.05 were considered significant.

**Supplementary figure 2**

There was no difference in the proportion of any CD4+ functional subsets that were T_CM_ except for RD-1-peptides-specific cells secreting IFN-γ only (top row) and TNF-α and IL-2 (bottom row). Patients with HIV co-infection (filled circles) and without HIV co-infection (open circles) are indicated. Results were analysed by Mann Whitney U test; and p values of <0.05 were considered significant.
