# Supplementary figures and images for: T-Cell Immunophenotyping Distinguishes Active From Latent Tuberculosis
Source: J Infect Dis. 2013 Sep 15;208(6):952–68. doi: 10.1093/infdis/jit265 (PMC3749005; doi:10.1093/infdis/jit265)

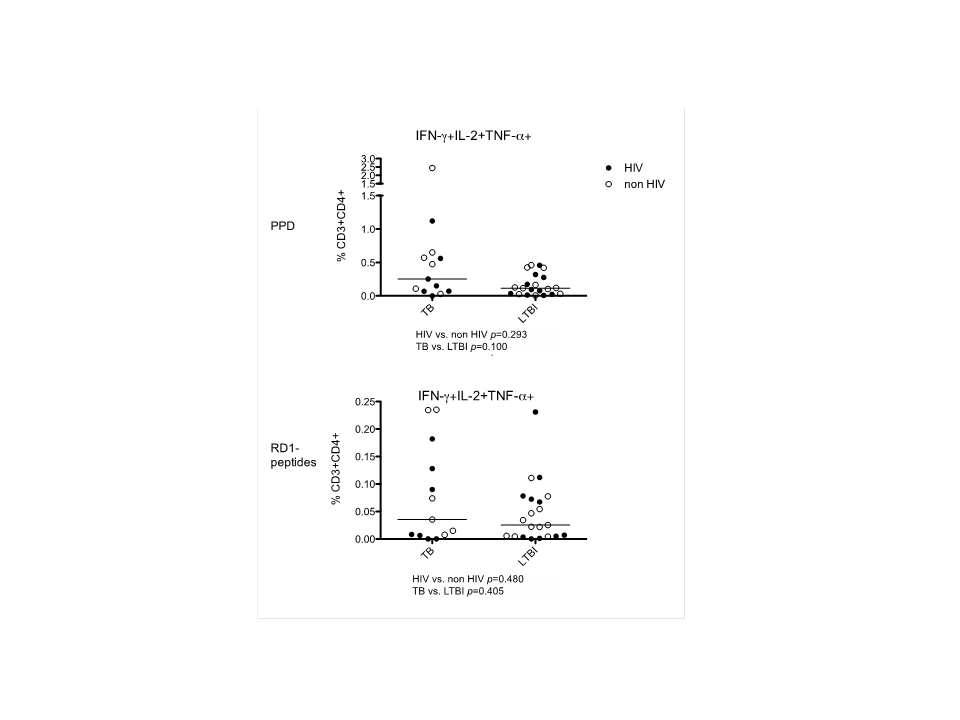

Supplement: Supplementary Data [file supp_jit265_jit265supp_fig1.tif]

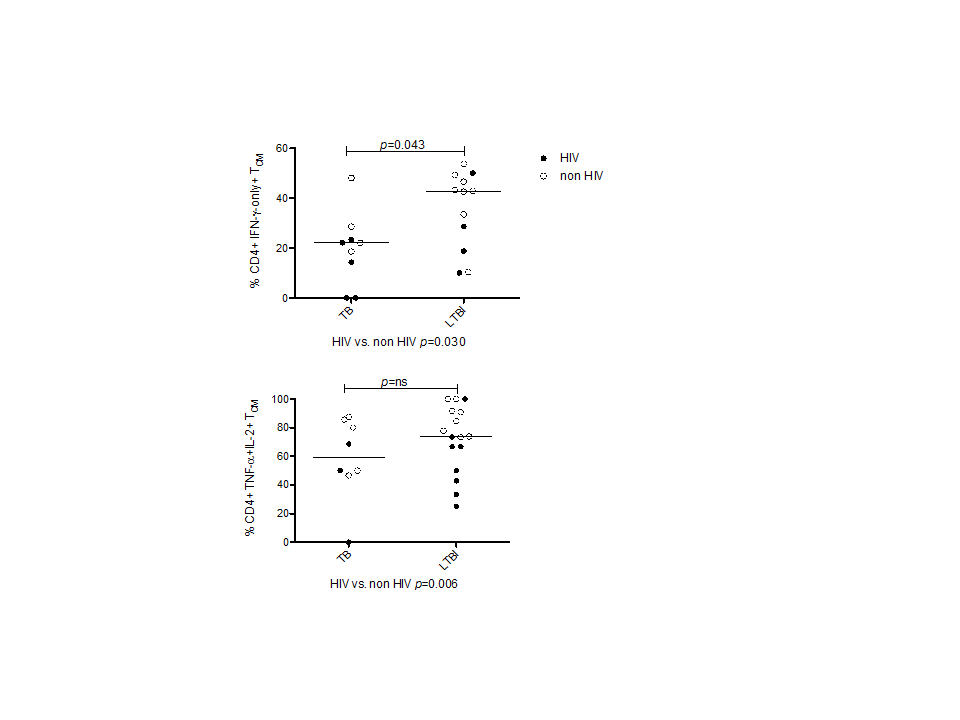

Supplement: Supplementary Data [file supp_jit265_jit265supp_fig2.tif]
